# A Purified Lycii Fructus Polysaccharide Fraction Extends Healthspan in *Caenorhabditis elegans* with an ATFS-1/UBL-5-Associated Mitochondrial Unfolded Protein Response

**DOI:** 10.3390/antiox15080944

**Published:** 2026-07-29

**Authors:** Yanting Hu, Xuhan Zhang, Yihang Xu, Wenhao Fan, Zhouyuan Xue, Yutong Wang, Zhongyuan Wang, Fang Zhang, Jialiang Hu, Zheng Qiu

**Affiliations:** 1 School of Life Science and Technology, China Pharmaceutical University, 639 Longmian Avenue, Nanjing 211198, China; 2 School of Pharmacy, Nanjing University of Chinese Medicine, 138 Xianlin Road, Nanjing 210023, China; fangzhang@njucm.edu.cn

**Keywords:** Lycii Fructus polysaccharide, healthspan, mitochondrial unfolded protein response, ATFS-1, *Caenorhabditis elegans*, human dermal fibroblasts

## Abstract

Mitochondrial unfolded protein response (UPR^mt^) is crucial in preserving mitochondrial health and, consequently, in prolonging healthspan. Natural bioactive polysaccharides have emerged as a central focus for delaying senescence, although their links to mitochondrial stress responses remain incompletely understood. In this study, a purified fraction of *Lycii Fructus* polysaccharide (FSP) significantly extended lifespan, improved healthspan-related phenotypes, and enhanced resistance to heat, oxidative, and ultraviolet stress in *C. elegans*. FSP also preserved mitochondrial abundance and morphology, increased adenosine triphosphate (ATP) levels and mitochondrial membrane potential, and reduced reactive oxygen species. Transcriptomic and qPCR analyses, together with *hsp-60* and *hsp-6* reporter assays, showed an enhanced UPR^mt^-associated response after FSP treatment. FSP-mediated lifespan extension was not observed in the *atfs-1* and *ubl-5* mutant strains, supporting pathway involvement of ATFS-1/UBL-5-dependent UPR^mt^ signaling. FSP reduced senescence-associated β-galactosidase positivity and improved mitochondrial function in human dermal fibroblasts. Our findings provide a vision of regulating UPR^mt^ for anti-aging interventions with plant polysaccharides and highlight the anti-aging potential of FSP by improving mitochondrial health.

## 1. Introduction

The emphasis on delaying aging has shifted in recent years, evolving from extending lifespan to prolonging healthspan [1], thereby enhancing overall quality of life and maintaining functional capacities during the later stages of life [2,3]. Among the various factors, mitochondria represent a particularly attractive focus for influencing healthspan [4,5].

Mitochondria are essential organelles responsible for cellular energy metabolism, generating ATP, and thereby influencing exercise capacity [4,6]. In addition, mitochondria are the primary sources and regulators of reactive oxygen species (ROS), which contribute significantly to the aging process and age-related diseases [7]. In addition to energy metabolism, mitochondria actively participate in cellular homeostasis through tightly regulated quality-control mechanisms [5,8]. Mitochondrial dysfunction is a hallmark of cellular senescence [9]. When mitochondrial function is compromised, a cascade of detrimental outcomes ensues, including reduced ATP synthesis, elevated ROS production, and impaired quality-control systems [7,8,10]. These disruptions not only accelerate cellular senescence but are also mechanistically associated with age-related declines, such as decreased exercise tolerance, accelerated muscle atrophy, and reduced healthspan [6,11].

Consequently, preserving mitochondrial health is critical for maintaining healthspan, including systemic metabolism, functional independence, and quality of life in later years [4,11]. One principal mechanism through which mitochondria support cellular and organismal health is the mitochondrial unfolded protein response (UPR^mt^) [12]. This evolutionarily conserved transcriptional program is activated in response to mitochondrial dysfunction and preserves mitochondrial proteostasis, mitigates environmental stress, and thereby maintains organismal health [13,14]. Studies have demonstrated that activation of UPR^mt^ can induce transcription of genes that maintain proteostasis [15], drive metabolic reprogramming [16], and coordinate stress-response pathways, positioning UPR^mt^ as a regulatory node linking mitochondrial stress signaling to longevity [13,14,16]. In an aging population, there is an increasing need for safe and effective interventions that target mitochondrial quality control and extend healthspan.

Some natural products have been reported to modulate mitochondrial homeostasis and UPR^mt^. Resveratrol enhances mitochondrial function by activating SIRT1/AMPK and reduces Aβ toxicity in *Caenorhabditis elegans*(*C. elegans)* via UBL-5-dependent UPR^mt^ [17,18]. Curcumin alleviates oxidative stress by inhibiting NF-κB and boosting antioxidant systems [19,20]. EGCG and quercetin have been shown to inhibit mitochondrial F0F1-ATPase/ATP synthase activity in isolated enzyme preparations [21]. Urolithin A enhances mitophagy and improves mitochondrial function, while spermidine induces mitochondrial quality control [22,23]. Trigonelline, an NAD^+^ precursor, stimulates respiration and biogenesis via sirtuins [24].

Among natural bioactive compounds, plant-derived polysaccharides have attracted increasing attention as potential interventions for age-related functional decline [25]. These macromolecules generally exhibit favorable biocompatibility and low toxicity, and can modulate multiple cellular pathways, a property well aligned with the multifactorial nature of aging [25,26,27]. Lycii Fructus, the dried ripe fruit of *Lycium barbarum* L. (Solanaceae), has been used as both a medicinal and dietary resource for centuries, and is associated with diverse health-promoting effects [28,29,30,31]. Accumulating evidence suggests that Lycii Fructus polysaccharides (LFPs) are among the bioactive components associated with these effects [32,33,34]. Despite extensive reports describing the antioxidant and cytoprotective properties of LFPs [31,34], the cellular stress-response pathways through which LFPs influence organismal aging remain incompletely defined. Given the central role of mitochondrial quality control in regulating healthspan and the emerging importance of UPR^mt^ as a longevity-associated stress response [13,16], we hypothesized that LFPs may modulate mitochondrial stress adaptation during aging.

To address this question, a purified LFP fraction, FSP, which was demonstrated to significantly extend the lifespan of Caenorhabditis elegans, was used in the current study. Structurally, FSP is composed of 33.4% linear homogalacturonan (HG) fragments and 54.5% rhamnogalacturonan-I (RG-I) sequences, with neutral side chains along the RG-I axis [35]. We systematically investigated its effects on aging phenotypes, mitochondrial integrity, and stress resilience in *C. elegans*. By integrating lifespan and healthspan analyses with transcriptomic profiling and genetic interrogation of key UPR^mt^ regulators, we assessed whether FSP-associated longevity involves UPR^mt^ signaling regulated by ATFS-1 and UBL-5.

## 2. Materials and Methods

### 2.1. C. elegans Strains and Maintenance

All *C. elegans* strains were maintained at 20 °C on standard nematode growth medium (NGM) agar plates seeded with *Escherichia coli* OP50 [36]. Unless otherwise stated, the following strains were obtained from the Caenorhabditis Genetics Center (CGC): N2 (wild-type); VC3201 *atfs-1* (gk3094) V; VC2654 *ubl-5* (ok3389) I; TK22 *mev-1* (kn1) III; SJ4103 zcIs14 [myo-3::GFP (mit)]; SJ4058 zcIs9 [*hsp-60*::GFP + lin-15 (+)] V; SJ4100 zcIs13 [*hsp-6*p::GFP + lin-15(+)] V; and PD4251 ccIs4251 [(pSAK2) myo-3p::GFP::LacZ::NLS + (pSAK4) myo-3p::mitochondrial GFP + dpy-20 (+)] I; dpy-20 (e1282) IV. The transgenic strain expressing *atfs-1*p::*atfs-1*::GFP was kindly provided by Dr. Ying Liu’s laboratory at Peking University (Beijing, China). Unless otherwise specified, FSP treatment was initiated at the synchronized L4 stage at 2 mg/mL, a concentration selected from the previous characterization of FSP [35]. OP50 was spread onto solid NGM agar plates and inactivated by UV irradiation for 15 min at a distance of 20 cm from the UV source to reduce potential effects of ongoing bacterial metabolism [36]. FSP was dissolved in purified water, and 200 μL of the FSP aqueous solution was added to the surface of NGM plates. Unless otherwise indicated, the number of *C. elegans* refers to the count per group. All experiments were performed with at least three biological replicates.

### 2.2. Lifespan Assays

Synchronized L4-stage worms were placed on NGM plates at 60 worms per group in each independent biological replicate. Worms were transferred to fresh plates daily, and deaths were recorded each day. Worms that failed to move after gentle stimulation with a platinum wire were scored as dead [37]. A minimum of 60 nematodes per group was used. Untreated worms served as the negative control; a pharmacological positive control for lifespan extension was not included. Worms that disappeared during the assay were right-censored on the last day on which they were observed, and were subsequently removed from the number at risk.

### 2.3. Head Swing and Pharyngeal Pumping Frequency Assay

Synchronized N2 worms were cultured on NGM plates. Head swinging was counted over 30 s intervals. Head swing observations were performed using an MSD202-B2 stereomicroscope (Murzider (Dongguan) Technology Co., Ltd., Dongguan, China) equipped with WF10×/20 mm wide-field eyepieces and a continuous total magnification range of 7–45× (6.4:1 zoom ratio), with a 100 mm working distance and independently adjustable upper and lower 12 V/15 W LED illumination. Worms that died from non-age-related causes were censored. Pharyngeal pumping frequency was measured by counting contractions of the pharynx over a 30 s interval [38]. Pharyngeal contractions were visualized using an M150i WiFi upright biological microscope (Motic, Xiamen, China) with integrated digital imaging (static resolution, 16 MP; dynamic resolution, 1080p) and MotiConnect software (version 1.4.9). Each experimental group contained at least 15 worms.

### 2.4. Body Length Measurement

Concurrently with the lifespan assay, NGM plates containing worms were imaged using an M150i WiFi upright biological microscope (Motic, Xiamen, China) with integrated digital imaging (static resolution, 16 MP; dynamic resolution, 1080p) and MotiConnect software on days 1, 3, 5, and 7. Each group comprised at least 15 worms. Body length was measured and analyzed using ImageJ software (version 1.54g) [39].

### 2.5. Fertility Assay

At least 10 synchronized L4-stage worms per condition were randomly transferred to fresh NGM plates (one worm per plate) and allowed to lay eggs for 24 h. Eggs were counted, and each parent was transferred to a fresh plate. These steps were repeated for 5 days. The total number of progeny produced during this period was recorded as fecundity [27].

### 2.6. Lipofuscin Quantification

Age-synchronized L4-stage worms were maintained on NGM plates with or without FSP. On days 5 and 11, at least 30 worms per group were randomly selected for lipofuscin analysis. Worms were mounted on slides and imaged using blue-light excitation (excitation, 400 nm; emission, 430 nm). Unless otherwise specified, fluorescence imaging in Section 2.6, Section 2.13, Section 2.14, Section 2.15, Section 2.17, Section 2.18 and Section 2.19 was performed using an Axio Observer Z1 fluorescence microscope (Carl Zeiss, Oberkochen, Germany) equipped with a 63×/1.4 NA Plan-Apochromat oil-immersion objective and an AxioCam MRm camera(Carl Zeiss Microscopy GmbH, Jena, Germany). Bright-field imaging of SA-β-gal-stained human dermal fibroblasts (HDFs) in Section 2.19 used the same microscope, objective, and camera. Identical acquisition settings were used for all groups within each experiment. Lipofuscin-associated autofluorescence intensity was quantified using ImageJ [40].

### 2.7. Stress Resistance Assays

Resistance to heat shock, oxidative stress, and UV irradiation was assessed as described previously, with minor modifications [37,38]. Approximately 30 worms were used per group in each independent biological replicate. For sustained heat stress, synchronized L4-stage worms were cultured for 3 days with or without FSP, transferred to fresh plates, and incubated at 35 °C. Survival was recorded hourly until all worms had died. For the heat-shock recovery assay, plates were incubated at 35 °C for 4 h and then returned to 20 °C for 12 h before survival was quantified. For oxidative stress, worms treated for 3 days were transferred to NGM plates containing 240 μM juglone and maintained at 20 °C; survival was assessed hourly until complete mortality. For UV stress resistance, synchronized L4-stage worms were pretreated with or without FSP for 3 days, transferred to fresh NGM plates, and exposed to a 22 W UV source for 10 min. Survival was monitored daily.

### 2.8. RNA Sequencing and Analysis

Synchronized L4-stage *C. elegans* were treated with 2 mg/mL FSP for 15 days in three independent biological replicates. After treatment, worms were washed and collected in sterile M9 buffer, and the pellets were rapidly frozen in liquid nitrogen. Transcriptome sequencing and differential gene-expression analysis were performed by Majorbio Bio-Pharm Technology Co., Ltd. (Shanghai, China).

Differentially expressed genes (DEGs) between the FSP-treated and control groups were identified using thresholds of |log_2_(fold change)| ≥ 1.5 and adjusted *p* < 0.05. Functional enrichment analyses were performed using the Gene Ontology (GO) and Kyoto Encyclopedia of Genes and Genomes (KEGG) databases. Bioinformatic analyses were conducted in part using the Majorbio Cloud platform [41]. The RNA-sequencing data are available in the NCBI Gene Expression Omnibus under accession GSE324598.

### 2.9. RNA Extraction and Quantitative RT-PCR

Approximately 1000 worms per condition were collected in M9 buffer for RNA extraction. Total RNA was isolated using TRIzol reagent (Invitrogen, Carlsbad, CA, USA). cDNA was synthesized using Hifair III 1st Strand cDNA Synthesis SuperMix (Yeasen Biotechnology, Shanghai, China). qPCR was performed on a QuantStudio 3 Real-Time PCR System (Applied Biosystems, Waltham, MA, USA) using the QuantiNova SYBR Green PCR Kit (Qiagen, Hilden, Germany). Primer sequences and corresponding gene information are provided in Table S1 [27].

### 2.10. Quantification of Mitochondrial GFP Fluorescence in C. elegans

The transgenic strain PD4251, ccIs4251 [(pSAK2) myo-3p::GFP::LacZ::NLS + (pSAK4) myo-3p::mitochondrial GFP + dpy-20(+)] I; dpy-20(e1282) IV, which expresses mitochondrially targeted GFP in body-wall and vulval muscle cells, was used to quantify relative mitochondrial GFP fluorescence [42]. Synchronized PD4251 worms were transferred at the L4 stage onto NGM plates with or without 2 mg/mL FSP. On days 5, 10, and 15 of adulthood, worms were washed from the culture plates and transferred to fresh NGM plates without OP50. At least 30 worms were analyzed per group at each time point. Worms were placed on agarose pads pre-spotted with 20 µL of levamisole hydrochloride solution, allowed to become fully paralyzed, and aligned for imaging. Fluorescence images were acquired using an Axio Observer Z1 fluorescence microscope (Carl Zeiss, Oberkochen, Germany) equipped with a 63×/1.4 NA Plan-Apochromat oil-immersion objective and an AxioCam MRm camera. GFP fluorescence was detected using a filter set with 470/40 nm excitation, 525/50 nm emission, and a 495 nm beam splitter. Relative mitochondrial GFP fluorescence was quantified using ImageJ (NIH, Bethesda, MD, USA).

### 2.11. Quantification of Mitochondrial DNA

Synchronized L4-stage *C. elegans* were treated with 2 mg/mL FSP for 5, 10, or 15 days. Genomic DNA was extracted using a genomic DNA extraction kit (Beyotime Biotechnology, Shanghai, China). Relative mitochondrial DNA copy number was estimated by qPCR as the ratio of a mitochondrial target to a nuclear reference target, following an established *C. elegans* procedure [43]. Primer sequences and corresponding gene information are provided in Table S1.

### 2.12. Measurement of ATP Levels

Intracellular ATP levels were quantified using an ATP assay kit (Beyotime Biotechnology, Shanghai, China), according to the manufacturer’s instructions. Worms were collected in M9 buffer and lysed using an equal volume of ATP-assay lysis buffer. ATP detection working solution (100 μL) was added and incubated in the dark for 5 min. Luminescence was measured immediately using a microplate luminometer. Relative light units were normalized to total protein concentration and are presented as fold changes relative to the control group. At least 15 worms were analyzed per group [44].

### 2.13. Reactive Oxygen Species Measurement

Overall intracellular ROS-associated fluorescence was measured using H2DCFDA (Beyotime Biotechnology, Shanghai, China). Because H2DCFDA responds to multiple intracellular oxidants, it was used as a non-mitochondria-specific readout of cellular oxidative status and was not interpreted as a selective measurement of mitochondrial ROS. Synchronized L4-stage worms were cultured on FSP-containing or control NGM plates. On days 4 and 10, worms were collected in M9 buffer and incubated with 50 μM H2DCFDA at 37 °C for 2 h in the dark with gentle shaking. Worms were then washed three times with M9 buffer, mounted on slides, and immobilized with 0.5 mM levamisole. Intestinal H2DCFDA-derived fluorescence was imaged using the setup specified in Section 2.6, with 492–495 nm excitation and 517–527 nm emission. At least 30 worms were analyzed per group, and fluorescence intensity was quantified in ImageJ using identical acquisition settings [45].

### 2.14. Mitochondrial Membrane Potential Assay

Mitochondrial membrane potential was assessed using MitoTracker Red CMXRos (Thermo Fisher Scientific, Waltham, MA, USA). A 1 mM stock solution was diluted and applied to bacterial lawns at a final concentration of 100 nM. Plates were sealed and incubated in the dark for 6 h. Worms were collected in M9 buffer, transferred to dye-treated plates, and incubated in the dark for 24 h. After immobilization with levamisole on agarose pads, fluorescence images were acquired using the microscope, objective, and camera specified in Section 2.6, with identical acquisition settings for all groups. At least 30 worms were analyzed per group, and fluorescence intensity was quantified using ImageJ [46].

### 2.15. Mitochondrial Network Dynamics Assessment

Mitochondrial network morphology was evaluated in SJ4103 (zcIs14 [myo-3::GFP(mit)]) worms. On days 5, 10, and 15, worms were randomly selected, mounted on slides, immobilized with 5 mM levamisole, and imaged using the fluorescence-imaging setup specified in Section 2.6. Mitochondrial morphology was classified according to established criteria [47]. Tubular mitochondria were interconnected and elongated; fragmented mitochondria were predominantly spherical or short; and intermediate morphology contained both interconnected and fragmented structures. Scoring was performed while blinded to treatment group, with at least 20 worms analyzed per group.

### 2.16. Mitochondrial Ultrastructure in C. elegans

Worms were fixed in electron-microscopy fixative at 4 °C and post-fixed with 1% osmium tetroxide in 0.1 M phosphate buffer (pH 7.4). After dehydration through a graded ethanol–acetone series, samples were infiltrated and embedded in Epon 812 resin and polymerized at 60 °C for 48 h. Ultrathin sections (60–80 nm) were prepared using an RMC PT-PC ultramicrotome (Boeckeler Instruments, Tucson, AZ, USA) equipped with an Ultra 45° diamond knife (Diatome, Nidau, Switzerland) and mounted on 150-mesh Formvar-coated copper grids. Sections were contrasted with uranyl acetate and lead citrate and examined using an HT7800 transmission electron microscope (Hitachi High-Tech, Tokyo, Japan) [48].

### 2.17. Quantification of hsp-6p::GFP and hsp-60::GFP Reporter Fluorescence

Transgenic strains SJ4100 (*hsp-6*p::GFP) and SJ4058 (*hsp-60*::GFP) were used to assess UPR^mt^ reporter activity. Fluorescence images were acquired using the microscope, objective, and camera specified in Section 2.6 with a GFP-compatible filter set (excitation, 488 nm; emission, 509 nm). Fluorescence intensity in the pharyngeal region was quantified using ImageJ [49]. Identical acquisition settings were used across groups, and at least 30 worms were analyzed per group.

### 2.18. ATFS-1::GFP Nuclear Translocation Assay

Nuclear translocation of ATFS-1::GFP was assessed in the transgenic *atfs-1*p::*atfs-1*::GFP strain. Synchronized L4-stage worms were cultured with or without FSP for 9 days and then exposed to 240 μM juglone for 1 h. GFP images were acquired using the fluorescence-imaging setup specified in Section 2.6, and ATFS-1 localization was quantified as the nuclear-to-cytoplasmic GFP fluorescence-intensity ratio [49].

### 2.19. Cell Culture and Treatments

Primary human dermal fibroblasts (HDFs) were obtained from Procell Life Science & Technology Co., Ltd. (Wuhan, China) and maintained in DMEM supplemented with 15% fetal bovine serum and 1% penicillin–streptomycin at 37 °C in a humidified incubator containing 5% CO_2_. FSP was dissolved in the culture medium and used at concentrations of 100 μg/mL and 200 μg/mL. For senescence-associated β-galactosidase (SA-β-gal) staining, HDFs were pretreated with FSP for 24 h and then co-incubated with 500 μM H_2_O_2_ for 2 h. The medium was subsequently replaced with FSP-containing medium, and cells were cultured for a further 72 h. SA-β-gal activity was assessed using the Senescence β-Galactosidase Staining Kit (Beyotime Biotechnology, Shanghai, China), according to the manufacturer’s instructions [50]. Bright-field images were acquired using the microscope, objective, and camera specified in Section 2.6. For mitochondrial function assays, HDFs were treated with or without FSP for 72 h. ATP levels, overall intracellular ROS-associated fluorescence, and mitochondrial membrane potential were measured as described in Section 2.12, Section 2.13 and Section 2.14, respectively; fluorescence images were acquired using the setup specified in Section 2.6.

### 2.20. Statistical Analysis

All experiments were performed independently at least three times. Unless otherwise stated, data are presented as the mean ± standard error of the mean (SEM). Statistical analyses were performed using GraphPad Prism 10.1.2. Some figures were generated using R 4.3.3. Survival curves were estimated using the Kaplan–Meier method and compared using the log-rank (Mantel–Cox) test. Two-group non-survival comparisons were performed using unpaired, two-tailed Student’s *t*-tests. Comparisons involving more than two groups were analyzed by one-way analysis of variance followed by the multiple-comparisons test specified in the corresponding figure legend. Statistical significance was defined as *p* < 0.05, * *p* < 0.05, ** *p* < 0.01, and *** *p* < 0.001. All *p* values < 0.001 are reported as *p* < 0.001 (***) regardless of the exact magnitude.

## 3. Results

### 3.1. FSP Improved Healthspan-Related Phenotypes and Multistress Resistance in C. elegans

**Figure 1:**
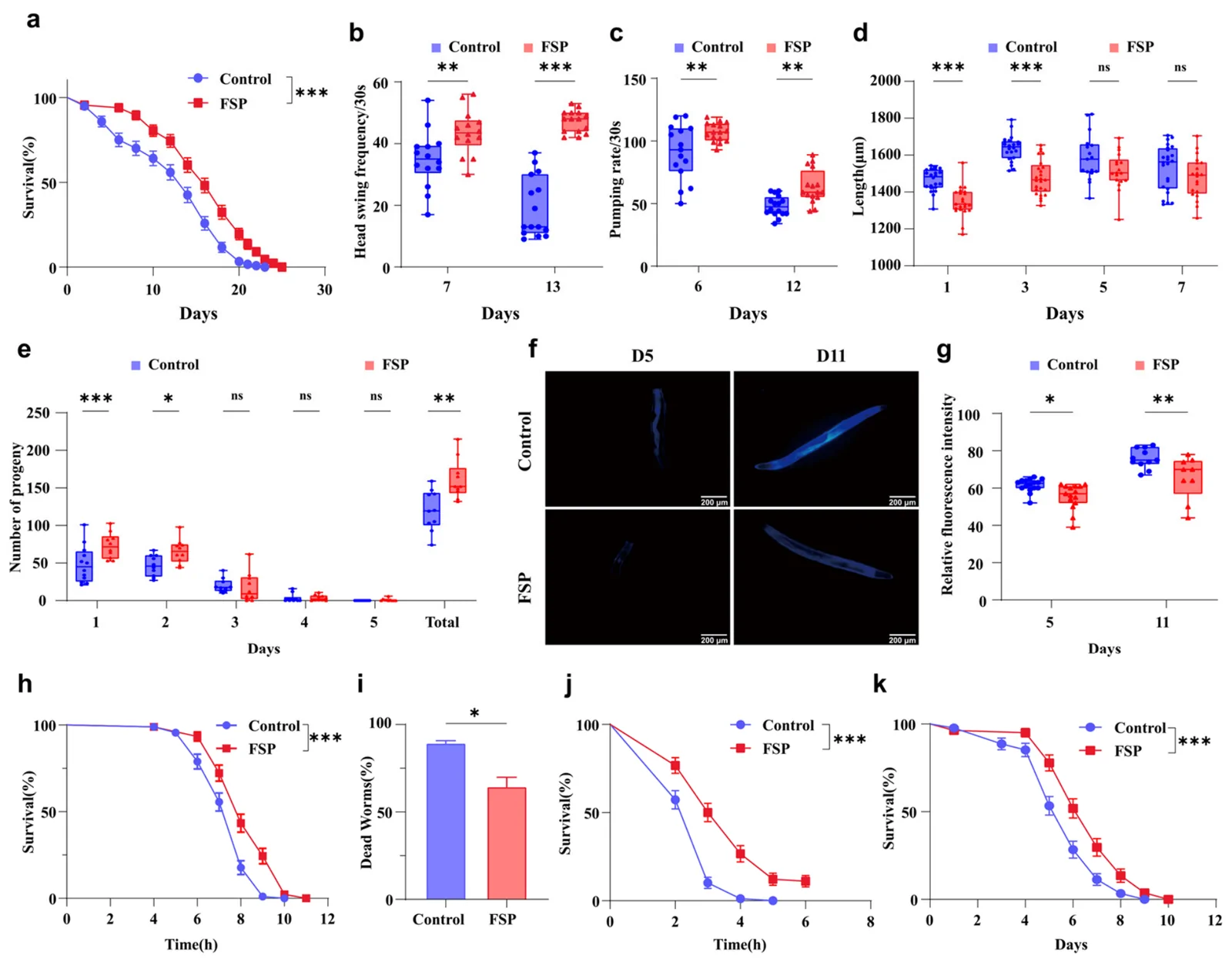
FSP extended the healthspan and stress resistance in wild-type *C. elegans*: (**a**) Survival curve of *C. elegans* treated with FSP under normal growth conditions. (**b**) Head swing frequency of N2 worms treated with or without FSP on days 7 and 13. (**c**) Pharyngeal pumping frequency of N2 worms treated with or without FSP on days 6 and 12. (**d**) Changes in body length of N2 worms treated with or without FSP. (**e**) Daily and total number of eggs laid by N2 worms treated with or without FSP. (**f**,**g**) Representative fluorescence images (**f**) and quantification of the fluorescence intensity (**g**) showing lipofuscin levels in N2 worms on days 5 and 11 with or without FSP treatment. (**h**) Survival curves of nematodes exposed to sustained 35 °C thermal stress. (**i**) Recovery assay: mortality quantification after 4 h 35 °C heat shock followed by 12 h recovery at 20 °C. (**j**) Survival curves under 240 μM juglone-induced oxidative stress after three-day FSP administration. (**k**) Survival curve following 10 min UV irradiation in FSP-pretreated worms. Survival curves in (**a**,**h**,**j**,**k**) were compared using the log-rank (Mantel–Cox) test. Other two-group comparisons were performed using unpaired, two-tailed Student’s *t*-tests at each indicated time point. * *p* < 0.05, ** *p* < 0.01, *** *p* < 0.001 indicate significant differences between groups.

Based on the previous characterization of FSP, *C. elegans* were cultured on UV-inactivated OP50-seeded NGM plates with or without 2 mg/mL FSP [35]. FSP treatment extended lifespan by 27.06% relative to the untreated control group (*p* < 0.001; Figure 1a). We next assessed aging-related physiological phenotypes. FSP attenuated age-associated declines in head swing frequency and pharyngeal pumping rate (Figure 1b,c). On day 13, the head swing frequency of FSP-treated worms increased by 143% compared with the controls (47.2 versus 19.4 bends per 30 s). FSP also altered body length during early adulthood, whereas this difference was not maintained at later time points (Figure 1d). Reproductive output was higher in the FSP-treated group, as indicated by progeny number (Figure 1e). Lipofuscin-associated autofluorescence was lower in FSP-treated worms than in age-matched controls on days 5 and 11 (Figure 1f,g).

We further assessed resistance to heat, oxidative, and UV stress [38]. Under sustained heat stress at 35 °C, FSP-treated worms showed a rightward shift in the survival curve relative to controls (Figure 1h). Following a 4 h heat shock and 12 h recovery period, cumulative mortality was lower in FSP-pretreated worms (Figure 1i). FSP treatment was also associated with increased survival during juglone-induced oxidative stress (Figure 1j) and after UV irradiation (Figure 1k), indicating improved resistance across the tested stress conditions. Detailed information on lifespan and stress resistance experiments is shown in Supplementary Table S2.

### 3.2. RNA-Seq Analysis Revealed Enrichment of Mitochondria-Associated Pathways in FSP-Treated Nematodes

**Figure 2:**
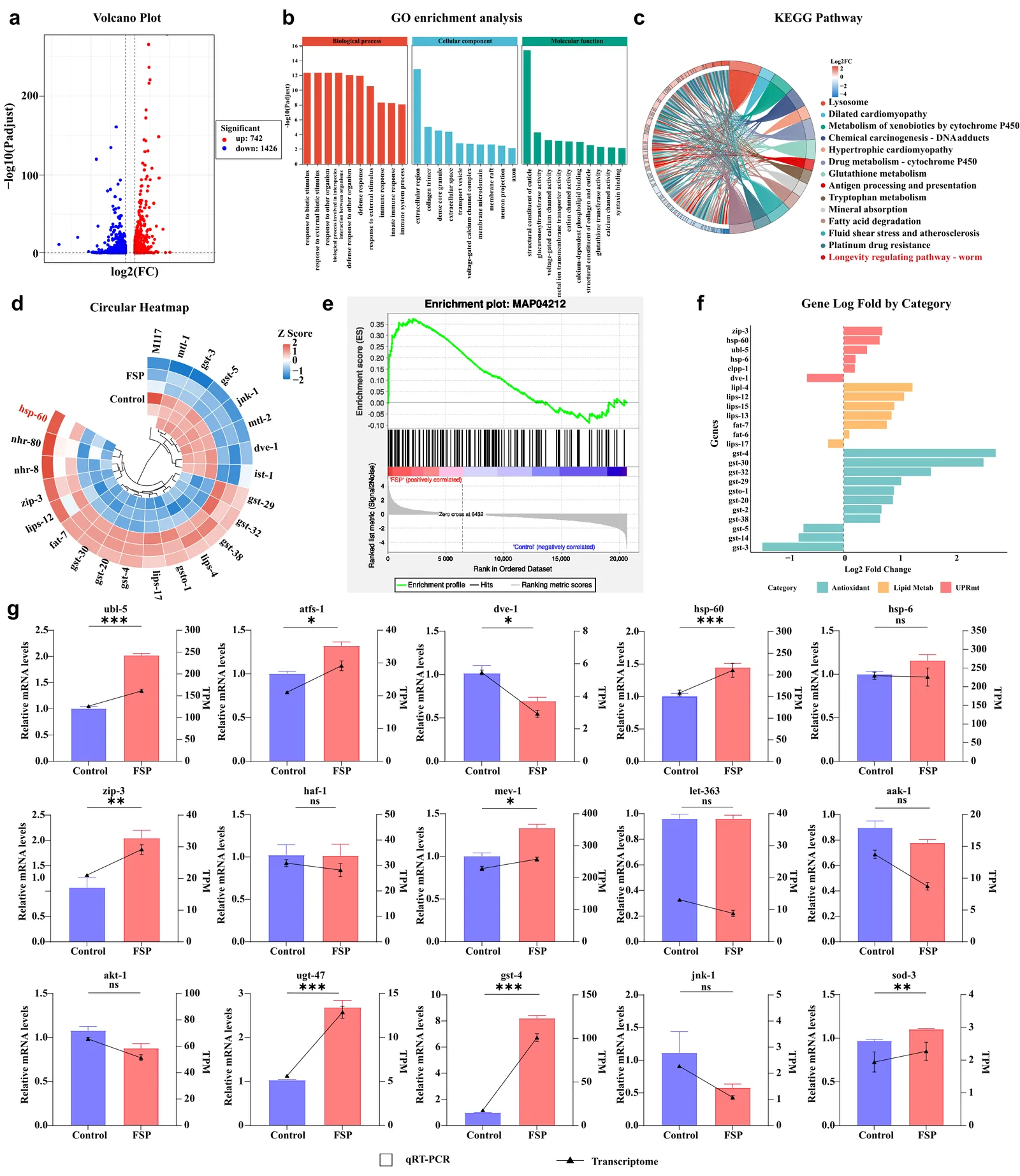
FSP extended nematode lifespan through mitochondria-associated pathways—differential expression analysis of genes between FSP-treated group and the control group: (**a**) Volcano plot of DEGs with thresholds set at |log_2_FC| ≥1.5 and adj.*p* value <0.05. Red dots indicate upregulated genes; blue dots indicate downregulated genes. (**b**) GO enrichment analysis of DEGs. (**c**) KEGG pathway enrichment analysis of DEGs. (**d**) Genes in the KEGG longevity-regulating pathway. (**e**) GSEA enrichment results for the longevity-regulating pathway. (**f**) Changes in selected genes from the KEGG longevity-regulating pathway. (**g**) The qPCR analysis of individual genes, compared with the transcriptomic data. qPCR comparisons were performed using unpaired, two-tailed Student’s *t*-tests. * *p* < 0.05, ** *p* < 0.01, *** *p* < 0.001 indicate significant differences between groups.

RNA-sequencing analysis identified 2168 DEGs (|log_2_ fold change| ≥ 1.5 and adjusted *p* < 0.05), including 742 upregulated and 1426 downregulated genes (Figure 2a). GO enrichment analysis associated these DEGs with responses to external stimuli, interspecies interactions, and immune-related processes (Figure 2b). KEGG enrichment analysis identified metabolic categories, including xenobiotic metabolism by cytochrome P450, glutathione metabolism, tryptophan metabolism, and fatty-acid degradation; the longevity-regulating pathway was also enriched (Figure 2c). Expression patterns of DEGs in the longevity-regulating pathway are shown in Figure 2d. Gene set enrichment analysis provided additional support for enrichment of the longevity-regulating pathway (Figure 2e). Selected genes associated with antioxidant responses, lipid metabolism, and UPR^mt^ signaling were further examined (Figure 2f). qPCR results were generally consistent with the transcriptomic data (Figure 2g): FSP increased the expression of *atfs-1*, *ubl-5*, *hsp-60*, *hsp-6*, *zip-3*, *haf-1*, and *mev-1*; decreased *let-363* and *aak-1* expression; and increased *ugt-47*, *gst-4*, and *sod-3* expression. These changes were consistent with altered mitochondrial stress-response and cellular-protection programs after FSP treatment.

### 3.3. FSP Preserved Mitochondrial Abundance and Function

**Figure 3:**
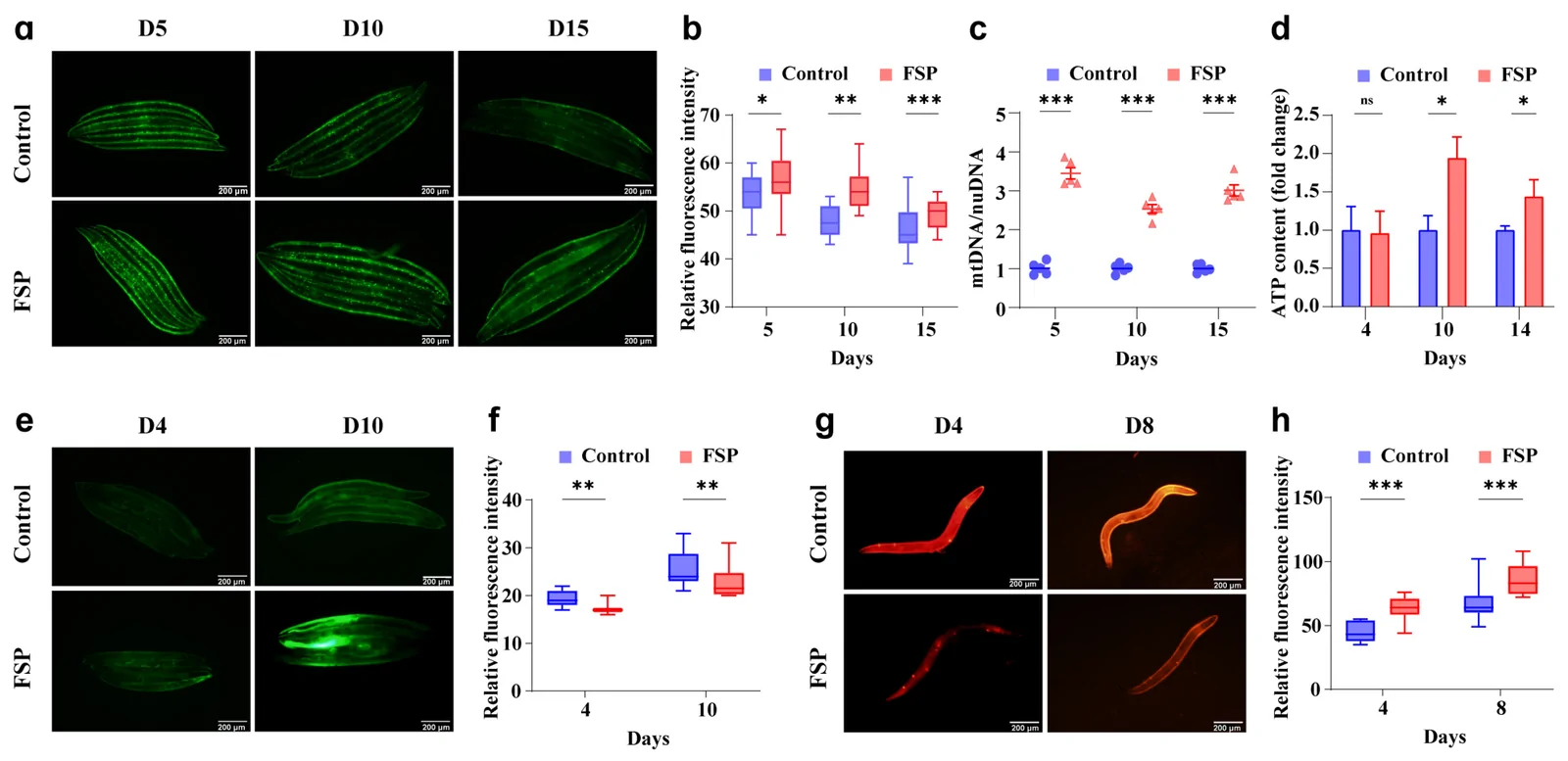
FSP improved mitochondrial abundance and function in *C. elegans*: (**a**,**b**) Representative fluorescence images (**a**) and quantification of the fluorescence intensity (**b**) of mitochondrial population in PD4251 worms on day 5, day 10, and day 15 with or without FSP treatment. (**c**) The mtDNA/nDNA ratio in control group and FSP-treated group. (**d**) ATP levels in control group and FSP-treated group. (**e**,**f**) Representative fluorescence images (**e**) of ROS accumulation quantification of the fluorescence intensity (**f**). (**g**) Representative fluorescence images of mitochondrial membrane potential (ΔΨm) and quantification of the fluorescence intensity (**h**). Two-group comparisons were performed using unpaired, two-tailed Student’s *t*-tests at each indicated time point. * *p* < 0.05, ** *p* < 0.01, *** *p* < 0.001 indicate significant differences between the groups.

PD4251 reporter worms were used to assess relative mitochondrial GFP fluorescence. FSP-treated worms showed higher mitochondrial GFP fluorescence than controls at early, middle, and late adult stages (Figure 3a,b). qPCR analysis also showed a higher mtDNA/nDNA ratio in the FSP-treated group at each assessed stage (Figure 3c), consistent with preservation of mitochondrial abundance. ATP levels were higher after FSP treatment, particularly at middle and late stages (Figure 3d). FSP reduced overall intracellular ROS-associated fluorescence measured using H2DCFDA (Figure 3e,f). In a separate assay, FSP attenuated the decline in mitochondrial membrane potential (ΔΨm; Figure 3g,h). Together, these measurements indicate lower overall cellular oxidant burden and improved mitochondrial functional status after FSP treatment.

### 3.4. FSP Maintained Mitochondrial Dynamics and Morphology

**Figure 4:**
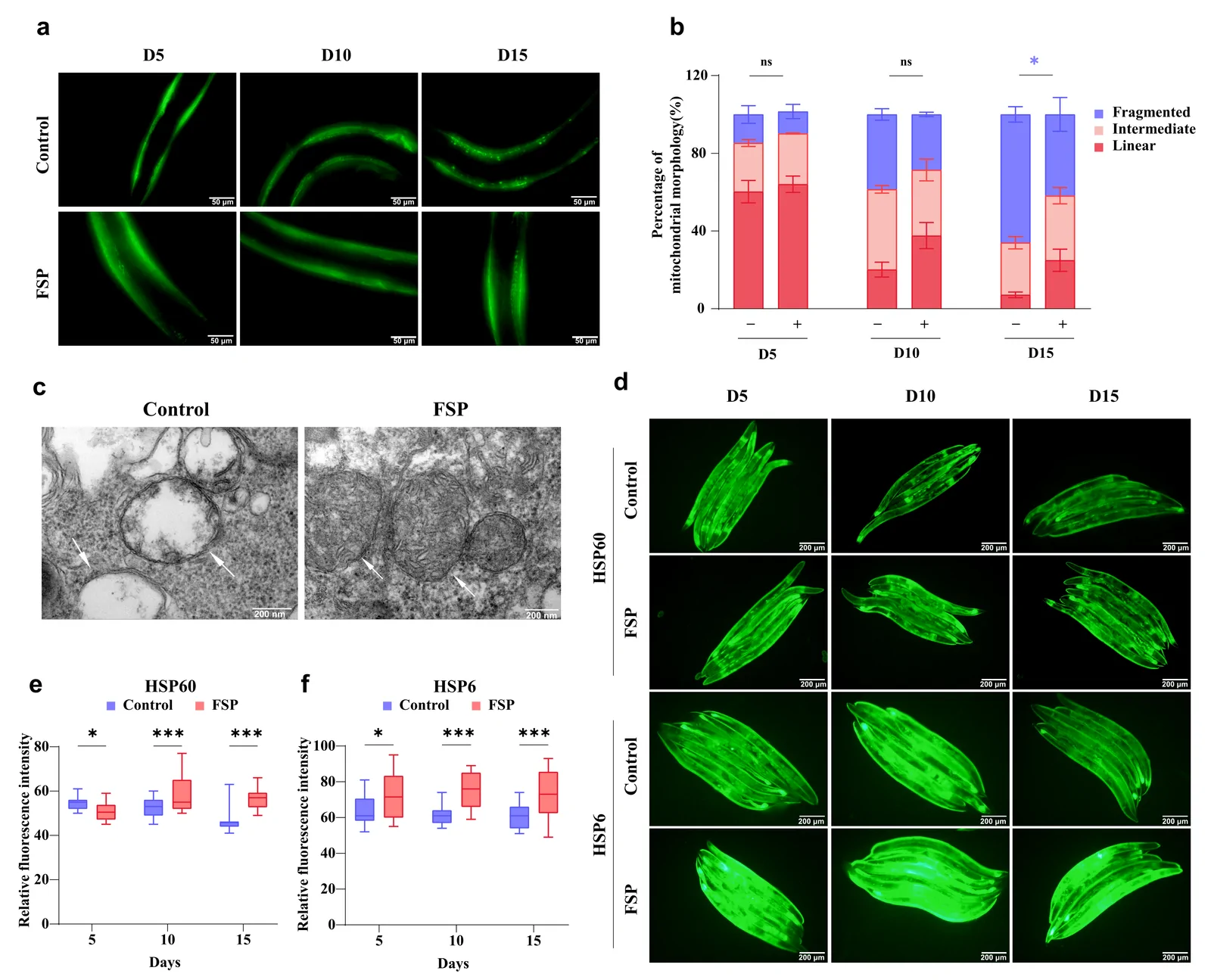
FSP preserved mitochondrial morphology and activated UPR^mt^ during aging in *C. elegans*: (**a**) Mitochondrial dynamics morphology of the control worm and the worm treated with FSP. (**b**) Quantification of mitochondrial morphology showing the proportions of tubular, intermediate, and fragmented mitochondria. (**c**) Transmission electron microscopy images illustrating mitochondrial ultrastructure in aged worms (day 15) with or without FSP treatment. The white arrows indicate mitochondria. (**d**) Representative fluorescence images of UPR^mt^ reporter strains SJ4058 (hsp-60::GFP) and SJ4100 (hsp-6p::GFP) at days 5, 10, and 15 under control and FSP-treated conditions. (**e**,**f**) Quantification of hsp-60::GFP (**e**) and hsp-6p::GFP (**f**) fluorescence intensity of nematodes with or without FSP treatment. Two-group comparisons were performed using unpaired, two-tailed Student’s *t*-tests at each indicated time point or morphology category. * *p* < 0.05, *** *p* < 0.001 indicate significant differences between the groups.

The SJ4103 reporter strain (zcIs14 [myo-3::GFP(mit)]) was used to evaluate mitochondrial network morphology in *C. elegans* [47]. On day 5, mitochondrial fragmentation did not differ significantly between control and FSP-treated worms. On days 10 and 15, control worms showed a lower proportion of tubular networks and a higher proportion of fragmented mitochondria, whereas FSP-treated worms retained a greater proportion of tubular structures. On day 15, tubular mitochondria accounted for 21.6% of scored networks in the FSP group and 9.8% in controls, while fragmented mitochondria accounted for 48.52% and 66.03%, respectively (Figure 4a,b). Transmission electron microscopy further showed that FSP-treated day-15 worms retained more clearly organized cristae than age-matched controls (Figure 4c).

### 3.5. FSP Was Associated with Increased HSP-60 and HSP-6 Reporter Signals During Aging

FSP treatment was associated with higher HSP-60 reporter fluorescence (Figure 4d). Relative to controls, mean fluorescence intensity in SJ4058 worms increased by 10.8% on day 10, and by 23.99% on day 15 (Figure 4d,e). SJ4100 worms also showed higher HSP-6 reporter fluorescence after FSP treatment at the middle and late stages, with increases of 21.43% on day 10 and 23.21% on day 15 (Figure 4d,f).

### 3.6. FSP-Associated Lifespan Extension Was Not Detected in the Tested atfs-1 and ubl-5 Mutants and Was Accompanied by OXPHOS-Related Effects in C. elegans

**Figure 5:**
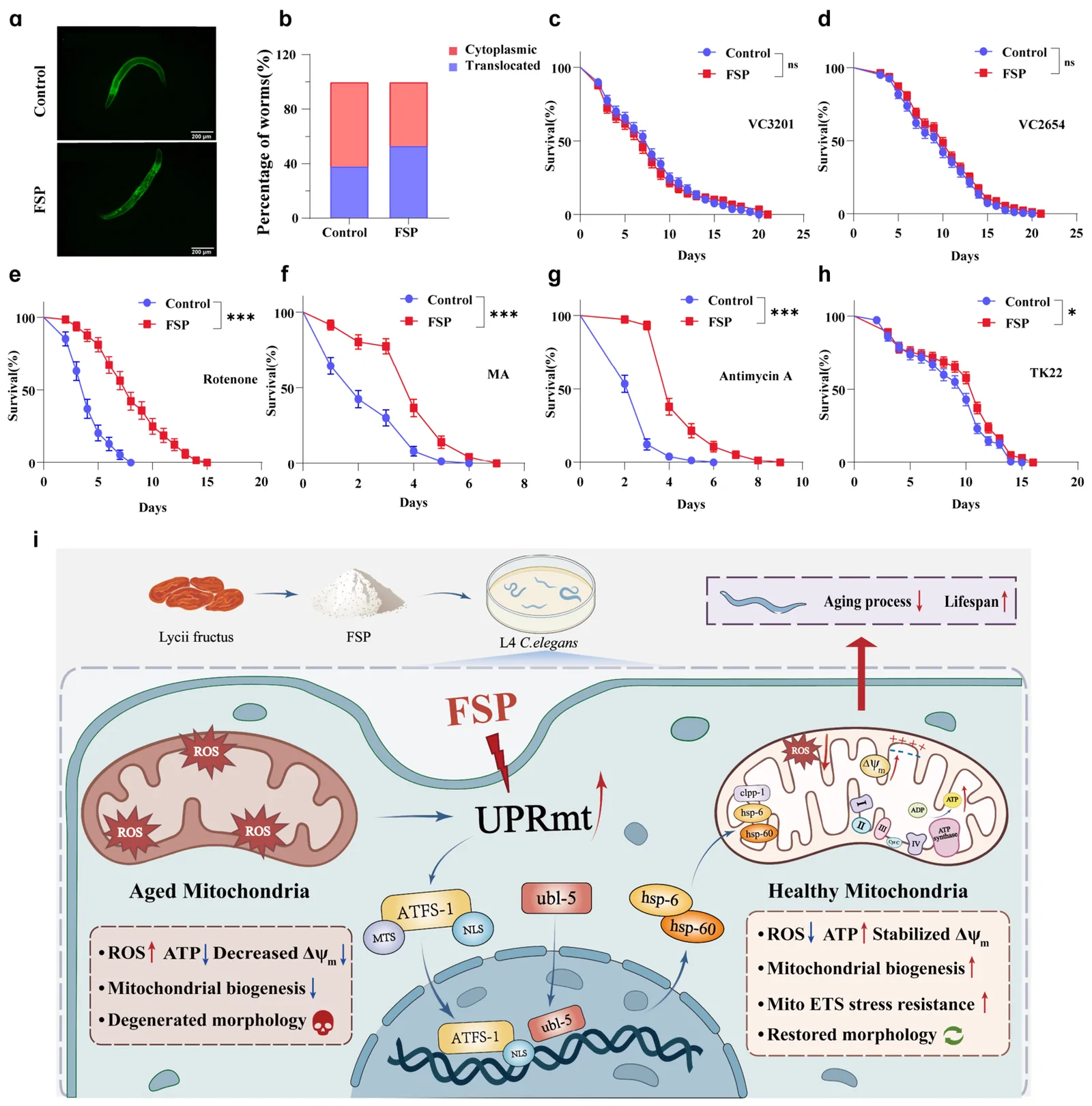
Genetic, reporter, and OXPHOS-related evidence for ATFS-1/UBL-5-associated UPR^mt^ involvement in the response to FSP: (**a**) Representative fluorescence images of ATFS-1 nuclear translocation under stress condition. (**b**) Proportions of translocated worms. (**c**) Survival curve of VC3201 *C. elegans* (atfs-1 mutant). (**d**) Survival curve of VC2654 *C. elegans* (ubl-5 mutant). (**e**) Survival curves of worms exposed to rotenone (complex I inhibitor) with or without FSP treatment. (**f**) Survival curves of worms exposed to malonate (MA; complex II inhibitor) with or without FSP treatment. (**g**) Survival curves of worms exposed to antimycin A (complex III inhibitor) with or without FSP treatment. (**h**) Survival analysis of the complex II–deficient mutant TK22 (*mev-1* strain) with or without FSP treatment. (**i**) The mechanisms of FSP-mediated longevity in *C. elegans*. Survival curves in (**c**–**h**) were compared using the log-rank (Mantel–Cox) test. * *p* < 0.05, *** *p* < 0.001 indicate significant differences between the groups.

Under juglone-induced oxidative stress, 53.13% of FSP-treated worms showed ATFS-1 nuclear localization, representing a 19.98% increase relative to the control group (Figure 5a,b). In the *atfs-1*(gk3094) mutant strain VC3201, the lifespan extension observed in wild-type worms was not detected (Figure 5c). The lifespan response was likewise not detected in *ubl-5*(ok3389) mutant worms (Figure 5d). The concordant findings for these two tested UPR^mt^ components, considered together with the reporter, qPCR, and ATFS-1 localization data, are consistent with pathway-level involvement of ATFS-1/UBL-5-associated signaling under the tested conditions.

To examine the relationship between the FSP response and respiratory-chain function, we used rotenone, malonate, and antimycin A to inhibit complexes I, II, and III, respectively. Each inhibitor shortened survival, while FSP treatment partially extended survival in inhibitor-exposed worms (Figure 5e–g). In TK22 *mev-1*(kn1) worms with impaired complex II function, the lifespan extension associated with FSP was attenuated relative to wild-type animals (9.23% versus 27.06%), although a residual response remained (Figure 1a and Figure 5h). ATFS-1 contributes to OXPHOS recovery by regulating mitochondrial and nuclear transcriptional programs [51,52]. The inhibitor and *mev-1* findings associate the response to FSP with partial maintenance of OXPHOS-related function, while the tested *atfs-1* and *ubl-5* mutant backgrounds are consistent with involvement of ATFS-1/UBL-5-associated UPR^mt^ signaling. Detailed information on the effects of FSP on lifespan under mitochondrial complex inhibition in wild-type *C. elegans* and in mutant *C. elegans* is provided in Tables S3 and S4, respectively.

The proposed model of FSP-mediated longevity in *C. elegans* is displayed in Figure 5i. During aging, mitochondria progressively lose number, structural integrity, and function, resulting in reduced ATP synthesis, elevated ROS levels, and decreased membrane potential. These impairments, in turn, accelerate aging and create a vicious cycle. FSP enhances the expression of ATFS-1 and promotes its translocation into the nucleus. Once in the nucleus, ATFS-1 upregulates the transcription of a series of UPR^mt^-related genes, including molecular chaperones (*hsp-60*), and, subsequently, regulates genes involved in OXPHOS (*mev-1*). This coordinated gene-expression program may help preserve mitochondrial integrity and function, as indicated by enhanced oxidative phosphorylation, restored mitochondrial membrane potential, attenuated ROS, promoted mitochondrial biogenesis, and a relatively preserved mitochondrial network. Collectively, these effects suggest a potential for extending the healthspan of *C. elegans*.

### 3.7. FSP Attenuated Senescence-Related Phenotypes and Supported Mitochondrial Function in HDFs

**Figure 6:**
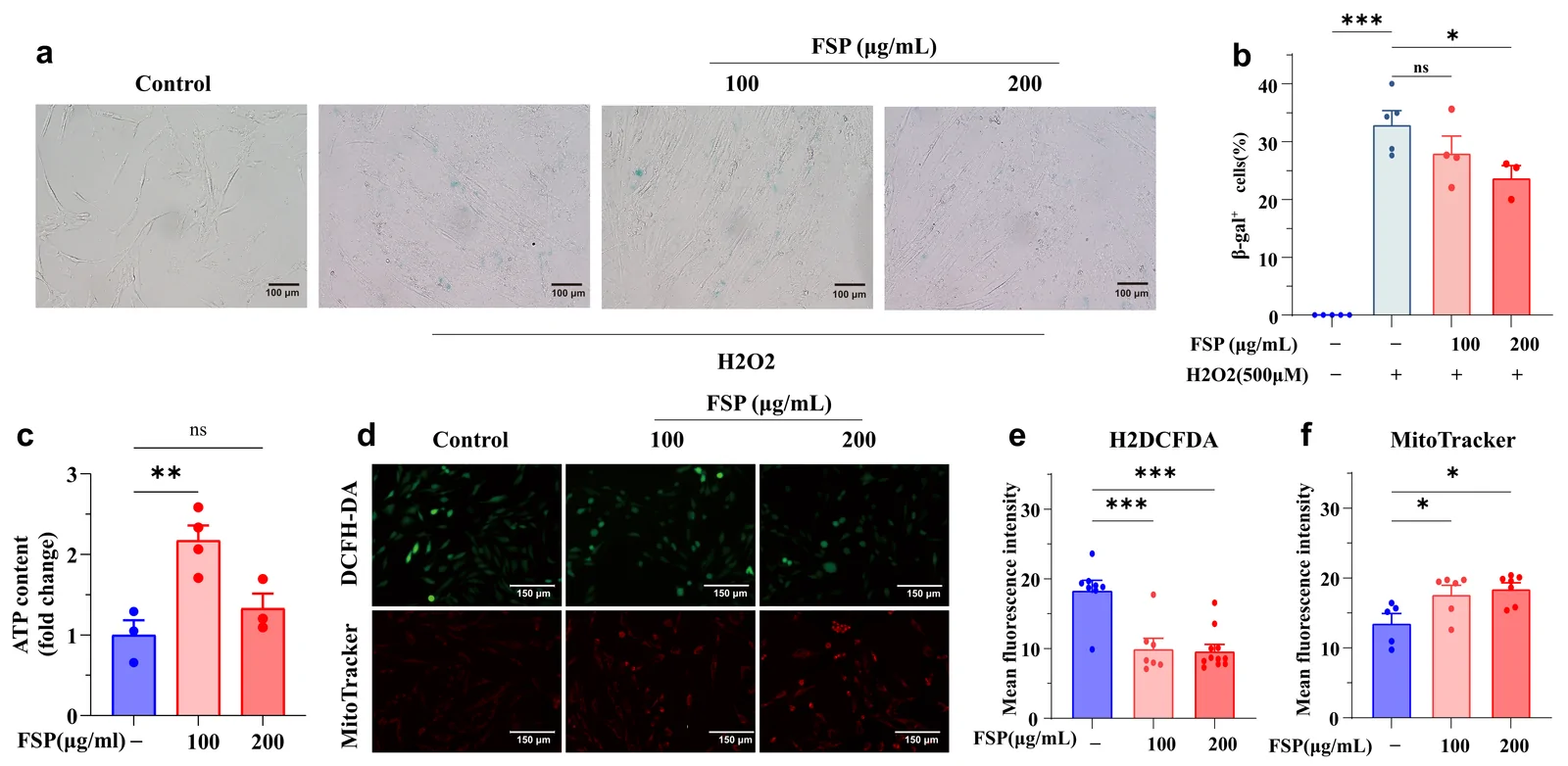
FSP mitigated senescence and promoted mitochondrial function in HDFs: (**a**) Representative image of SA-β-gal staining. (**b**) Quantification of SA-β-gal staining. (**c**) ATP levels in control group and FSP-treated group. (**d**) Representative fluorescence images of ROS accumulation (upper panel) and mitochondrial membrane potential (lower panel). (**e**) Quantification of ROS accumulation. (**f**) Quantification of mitochondrial membrane potential (ΔΨm). Comparisons involving more than two groups were performed using one-way ANOVA followed by Tukey’s multiple-comparisons test. * *p* < 0.05, ** *p* < 0.01, and *** *p* < 0.001, indicate significant differences between the groups.

HDFs were used to assess whether the cellular effects observed in worms were accompanied by related changes in a human cell model. H_2_O_2_ exposure increased the proportion of SA-β-gal-positive cells. Treatment with 200 μg/mL FSP reduced SA-β-gal positivity relative to the H_2_O_2_ group (*p* < 0.05), while the reduction at 100 μg/mL was not statistically significant (Figure 6a,b). FSP treatment was also associated with higher ATP levels (Figure 6c), lower overall intracellular H2DCFDA-associated fluorescence (Figure 6d,e), and higher mitochondrial membrane potential (Figure 6d,f). H2DCFDA fluorescence was interpreted as an overall readout of intracellular oxidants. These findings provide preliminary evidence that FSP attenuates senescence-related phenotypes and supports mitochondrial functional status in this HDF model.

## 4. Discussion

In our preliminary studies, crude polysaccharides were obtained from *Lycium barbarum* via water extraction and alcohol precipitation. To reduce potential interference from co-extracted components during biological activity assessment, the crude extract was further fractionated by Fehling precipitation into three distinct fractions (FP, FSP, and FSS). These fractions showed structure-dependent differences in their effects on both lifespan and gut microbiota composition. Among them, FSP demonstrated the most pronounced lifespan-extending effect under the tested conditions [35]. The present work expands this framework by establishing a mechanistic link between the purified FSP fraction and the maintenance of mitochondrial homeostasis.

Within natural products, mitochondrial adaptation has been associated with several complementary routes, including SIRT1/AMPK signaling, mitophagy, and NAD^+^-linked bioenergetics [17,22,23,24]. Our observations showed that FSP extended the healthspan of *C. elegans* and promoted mitochondrial function. At the mechanistic level, FSP treatment increased expression of UPR^mt^-related genes and induced greater ATFS-1 nuclear localization. ATFS-1 localization is regulated by mitochondrial import efficiency and coordinates OXPHOS recovery through mitochondrial and nuclear transcriptional programs [51,52]. The lifespan extension was not detected in the *atfs-1* (gk3094) and *ubl-5* (ok3389) mutants. Together with the reporter and transcriptional data, these findings suggested an ATFS-1/UBL-5-associated UPR^mt^ pathway. The partial response retained in *mev-1* mutants and respiratory-chain inhibitor experiments further associates this stress program with OXPHOS maintenance and mitochondrial adaptation.

To evaluate the translational relevance of our findings, we extended the investigation to HDFs as a mammalian cell model. Treatment with FSP significantly reduced the intracellular oxidant burden, as indicated by attenuated SA-β-gal staining and concurrently decreased ROS, elevated ATP production, and restored mitochondrial membrane potential. These functional improvements closely parallel the mitochondrial protective effects observed in *C. elegans.* Mammalian UPR^mt^ signaling involves ATF5 and related stress regulators [15], and this conservation provides a molecular basis for further mechanistic studies in mammalian systems.

Several limitations should be considered when interpreting these findings. The genetic analysis used one allele each for the *atfs-1* and *ubl-5* mutants; independent alleles, RNAi-based knockdown, and genetic rescue would strengthen the evidence for their involvement. Although our study suggests that FSP modulates UPR^mt^, the precise molecular mechanisms governing this interaction remain to be elucidated. Polysaccharides are complex macromolecules, and their mode of action can be multifaceted. It remains unclear whether FSP directly targets UPR^mt^ signaling molecules, exerts its effects through degradation products, or regulates the UPR^mt^ indirectly by modulating the gut microbiota and subsequent microbial-derived metabolites. It represents a compelling avenue for future research.

## 5. Conclusions

Using *C. elegans* as a model, this study showed that FSP treatment improved both lifespan and healthspan while conferring resistance to multiple forms of stress. FSP treatment was associated with preserved mitochondrial abundance, network integrity and morphology, higher ATP production, maintenance of membrane potential, and lower overall intracellular ROS. Genetic and reporter data are consistent with involvement of ATFS-1/UBL-5-associated UPR^mt^ signaling and OXPHOS-related function. Preliminary findings in HDFs were consistent with attenuation of senescence-related phenotypes and improved mitochondrial functional readouts. In summary, these findings highlight the health benefits of FSP and underscore its potential application in anti-aging therapies through the improvement of mitochondrial function.

## Data Availability

The RNA-sequencing datasets generated in this study are available in the NCBI Gene Expression Omnibus under accession no. GSE324598. Processed gene-expression matrices are included in the GEO submission. Other data supporting the findings are available from the corresponding author upon reasonable request.

## Supplementary Materials

The following supporting information can be downloaded at: https://www.mdpi.com/article/10.3390/antiox15080944/s1, Table S1: List of forward and reverse primers used in quantitative RT-PCR assays for gene expression and mtDNA/nDNA ratio; Table S2: Effects of FSP on lifespan and stress resistance in wild-type *C. elegans*; Table S3: Effects of FSP on lifespan under mitochondrial complex inhibition in wild-type *C. elegans*; Table S4: Effects of FSP on lifespan in mitochondrial mutants of C. elegans.

## Abbreviations

The following abbreviations are used in this manuscript:

ROS	Reactive oxygen species
UPR^mt^	Mitochondrial unfolded protein response
ΔΨm	Mitochondrial membrane potential
ATP	Adenosine triphosphate
LFPs	Lycii Fructus polysaccharides
HG	Homogalacturonan
RG-I	Rhamnogalacturonan-I
NGM	Nematode growth medium
HDFs	Human dermal fibroblasts
SA-β-gal	Senescence-associated β-galactosidase
HSP-6	Heat shock protein 6
HSP-60	Heat shock protein 60
ATFS-1	Activating transcription factor associated with stress-1
UBL-5	Ubiquitin-like protein 5
OXPHOS	Oxidative phosphorylation
