# Supplementary material for: A Purified Lycii Fructus Polysaccharide Fraction Extends Healthspan in Caenorhabditis elegans with an ATFS-1/UBL-5-Associated Mitochondrial Unfolded Protein Response
Source: Antioxidants (Basel). 2026 Jul 29;15(8):944. doi: 10.3390/antiox15080944 (PMC13509568; doi:10.3390/antiox15080944)
Supplement: Supplementary file 1 [file antioxidants-15-00944-s001.zip › antioxidants-4421896-supplementary.pdf]

Supporting Information

A Purified Lycii Fructus Polysaccharide Fraction Extends Healthspan in *Caenorhabditis elegans* with an ATFS-1/UBL-5-Associated Mitochondrial Unfolded Protein Response

Table S1 List of forward and reverse primers used in quantitative RT-PCR assays for gene expression and mtDNA/nDNA ratio.

Gene	*Gene ID*	*Direction*	*Sequence (5′–3′)*	*Product size(bp)*
act-3	WBGene00000065	Forward primer	TGCGACATTGATATCCGTAAGG	59
		Reverse primer	GGTGGTTCTCCGGAAAGAA	
nd-1	WBGene00010959	Forward primer	AGCGTCATTTATTGGGAAGAAGAC	75
		Reverse primer	AAGCTTGTGCTAATCCCATAAATGT	
act-1	WBGene00000063	Forward primer	CTACGAACTTCCTGACGGACAAG	102
		Reverse primer	CCGGCGGACTCCATACC	
ubl-5	WBGene00006726	Forward primer	ATGATTGAAATCACAGTAAA	222
		Reverse primer	TCATTGGTAGTAGAGCTCGA	
atfs-1	WBGene00013878	Forward primer	CAATCACCATCAAAATCGGCG	115
		Reverse primer	CTTGCTCAATGTCCATTTCGAAC	
dve-1	WBGene00022861	Forward primer	GCAGGGACCACTACATTAAATCA	124
		Reverse primer	GATCGAATGGCAAGGGTTTCC	
hsp-60	WBGene00002025	Forward primer	AGGGATTCGAGAGCATTCGTCAAG	143
		Reverse primer	TGTGGCGACTTGAGCGATCTCTTC	
hsp-6	WBGene00002010	Forward primer	GGAACAACAGATCGTTATCCAATC	153
		Reverse primer	GTGTCGTGGATGATTCCTTCAG	
zip-3	WBGene00012330	Forward primer	CGCACCCGATTCCAGTTGATAC	107
		Reverse primer	CATTCCAGATGATGAGGATTGTGG	
haf-1	WBGene00001811	Forward primer	TTGGAACCGTTGGTCAGGAG	402
		Reverse primer	CCACTTGGTCAGCTTGTTGC	
mev-1	WBGene00003225	Forward primer	ATCGATCGTCACCAAGTCCG	146
		Reverse primer	GGAATCCGGAGAGCATCCAG	
let-363	WBGene00002583	Forward primer	CCCACAATGCACTAAATCGGC	112
		Reverse primer	CGATTGGAAGTTGTGGCTGC	
aak-1	WBGene00019801	Forward primer	TGCATAGAGACTTGAAGCCCG	77
		Reverse primer	CGAGAGACCGAAATCAGCGA	
akt-1	WBGene00000102	Forward primer	AGTCGGCAGAAGTTCGTCAG	183
		Reverse primer	CGTGTCCCGAAGATGGTTGA	
ugt-47	WBGene00011006	Forward primer	TTCCCCCGCAACTAGCAAAT	71
		Reverse primer	GCCCAGCTTTTGCCAATTCA	
gst-4	WBGene00001752	Forward primer	CTCTTGCTGAGCCAATCCGT	113
		Reverse primer	CTGGCCAAATGGAGTCGTTG	
jnk-1	WBGene00002178	Forward primer	TGTGCACATGGAGGAACGATT	175
		Reverse primer	TGGAACCAGCCAATTCCCAA	
sod-3	WBGene00004932	Forward primer	TCTACTGCTCGCACTGCTTC	79
		Reverse primer	CTGGGAGAGTGTGCTTGGAG	

Table S2. Effects of FSP on lifespan and stress resistance in wild-type *C. elegans.*

Each row summarizes data from one independent lifespan or stress resistance experiment performed at 20 °C on standard NGM plates seeded with OP50. FSP was administered at 2 mg/mL in the culture medium. Stress conditions: heat shock at 35 °C (continuous exposure), oxidative stress with 240 μM juglone, and UV irradiation at 8 W for 10 min. N

Strain	Stress	Dose	Treatment	Replicates (n)	N (total/censored)	Mean lifespan	% Change (mean)	Median lifespan	% Change (median)	Statistical test	p-value
N2	none		Control	3	180/60	12.49 days		14 days			
N2	none		FSP (2 mg/mL)	3	180/47	15.89 days	27.22	17 days	21.43	Kaplan-Meier	p <0.0001
N2	Heat	35 °C	Control	3	90/0	7.97 hrs		8 hrs			
N2	Heat	35 °C	FSP (2 mg/mL)	3	90/0	8.83 hrs	10.79	8 hrs	0.00	Kaplan-Meier	p <0.0001
N2	Oxidative	240 μM Juglone	Control	3	90/1	3.18 hrs		3 hrs			
N2	Oxidative	240 μM Juglone	FSP (2 mg/mL)	3	90/0	4.09 hrs	28.61	3.5 hrs	16.67	Kaplan-Meier	p <0.0001
N2	UV	8W for 10 min	Control	3	90/2	6.15 days		6 days			
N2	UV	8W for 10 min	FSP (2 mg/mL)	3	90/9	7.12 days	15.77	7 days	16.67	Kaplan-Meier	p <0.0001

represents the total number of worms scored; censored worms (those lost due to bagging, bursting, or other non-aging causes) are indicated. Mean and median lifespans are presented as days (or hours) of adulthood; percentage changes are calculated relative to the corresponding untreated control. Statistical significance was assessed by the Kaplan-Meier log-rank test, with *p* < 0.05 considered significant. All experiments were repeated at least three times.

Table S3. Effects of FSP on lifespan under mitochondrial complex inhibition in wild-type *C. elegans.*

Strain	Stress	Dose	Treatment	Replicates (n)	N (total/censored)	Mean lifespan	% Change (mean)	Median lifespan	% Change (median)	Statistical test	p-value
N2	Rotenone	10 μM	Control	3	75/21	6.67 days		4 days			
N2	Rotenone	10 μM	FSP (2 mg/mL)	3	75/15	13 days	94.90-	8 days	100.00	Kaplan-Meier	p <0.0001
N2	MA	30 mM	Control	3	90/17	2.74 days		2 days			
N2	MA	30 mM	FSP (2 mg/mL)	3	90/19	4.04 days	45.99	4 days	100.00	Kaplan-Meier	p <0.0001
N2	Antimycin A	40 μM	Control	3	90/17	4 days		3 days			
N2	Antimycin A	40 μM	FSP (2 mg/mL)	3	90/16	7.33 days	83.25	4 days	33.33	Kaplan-Meier	p <0.0001

Worms were exposed to the following mitochondrial complex inhibitors: rotenone (Complex I, 10 μM), malonate (Complex II, 30 mM), and antimycin A (Complex III, 40 μM).

All assays were performed at 20 °C on NGM plates with or without 2 mg/mL FSP. N is the total number of worms; censored individuals are excluded from median and mean calculations. Lifespan values are given in days of adulthood. Percentage changes refer to the respective control groups without FSP. The Kaplan-Meier log-rank test was used to determine *p*-values; *p* < 0.05 was considered statistically significant. Each condition was tested in three independent replicates.

Table S4. Effects of FSP on lifespan in mitochondrial mutants of *C. elegans*.

**Strain**	Genotype	**Treatment**	**Replicates (n)**	**N (total/censored)**	**Mean lifespan**	**% Change (mean)**	**Median lifespan**	**% Change (median)**	**Statistical test**	**p-value**
VC3201	atfs-1(gk3094) V	Control	3	180/31	9.28 days		8 days			
VC3201	atfs-1(gk3094) V	FSP (2 mg/mL)	3	180/23	9.76 days	5.17	7 days	-12.5	Kaplan-Meier	p =0.9837
VC2654	ubl-5(ok3389) I	Control	3	180/16	10.21days		10 days			
VC2654	ubl-5(ok3389) I	FSP (2 mg/mL)	3	184/0	10.87 days	6.46	10 days	0.00	Kaplan-Meier	p =0.1171
TK22	mev-1(kn1) III	Control	3	180/38	9.84 days		10 days			
TK22	mev-1(kn1) III	FSP (2 mg/mL)	3	180/24	10.65 days	8.23	11 days	9.09	Kaplan-Meier	p =0.0225

Lifespan assays were carried out on mutant strains lacking key UPR^{mt} regulators: *atfs-1(gk3094)* (VC3201), *ubl-5(ok3389)* (VC2654), and *mev-1(kn1)* (TK22). Worms were maintained at 20 °C on NGM plates with or without 2 mg/mL FSP. N indicates the total number of worms scored per experiment; censored worms (due to non-aging death or technical loss) are shown in parentheses. Mean and median lifespans are reported in days of adulthood; percentage changes are calculated relative to the control group of the same genotype. Statistical analysis was performed using the Kaplan-Meier log-rank test, with significance set at *p* < 0.05. Results show that FSP-mediated lifespan extension is fully dependent on *atfs-1* and *ubl-5*, while the effect is partially preserved in *mev-1* mutants. Each experiment was performed in triplicate.
